# Triple M Overlap Syndrome: A Rare Complication of Immune Checkpoint Inhibitors

**DOI:** 10.7759/cureus.115859

**Published:** 2026-09-06

**Authors:** Brian Morris

**Affiliations:** 1 Medicine, University of California, Los Angeles (UCLA), Los Angeles, USA

**Keywords:** cancer immunotherapy, hepatic tumors, immune therapy mediated myocarditis, inflammatory myositis, myasthenic crisis

## Abstract

Immune checkpoint inhibitors are a major part of many cancer treatment protocols. Most side effects are generally minor, including fatigue, rashes, flu-like symptoms, headaches, or cough. However, some potential side effects may require prompt treatment to ensure optimal outcomes, so it is essential for clinicians to be knowledgeable about these rare but possible complications. We report a case of an 88-year-old woman who presented with the triple M overlap syndrome (TMOS) of myasthenia gravis, myositis, and myocarditis after receiving four rounds of atezolizumab treatment. This diagnosis was confirmed based on the elevated acetylcholine level of 0.89 nmol/L, elevated C-reactive protein level of 1.0 mg/dL, and peak troponin level of 1011 pg/mL. She was treated with high-dose steroids as well as intravenous immunoglobulins and showed clinical improvement. She was eventually transferred to a rehab facility.

## Introduction

Immune checkpoint inhibitors (ICIs) have revolutionized the care of cancer patients in recent years. These medications are generally well-tolerated by most patients [1]. However, there are risks associated with such treatments that can be significant and even potentially life-threatening [1,2]. Accordingly, clinicians must be aware of potential side effects, as these complications may require prompt treatment to ensure optimal outcomes [3]. Herein, we present a case of an 88-year-old woman with advanced hepatocellular carcinoma who received two cycles of atezolizumab and bevacizumab and then developed the so-called triple M overlap syndrome (TMOS).

## Case presentation

An 88-year-old woman with a history of breast cancer, hyperlipidemia, hypothyroidism, and gastroesophageal reflux disease (GERD) presented with abnormal liver function test (LFT) results. Computerized tomography (CT) imaging of the abdomen revealed a 15 cm hepatic mass, and further testing was consistent with hepatocellular carcinoma stage IV. The patient was evaluated by a general surgeon and deemed a non-surgical candidate due to the size and location of the tumor, which was pushing the diaphragm into the chest cavity as seen on chest X-ray (Figure 1).

**Figure 1:**
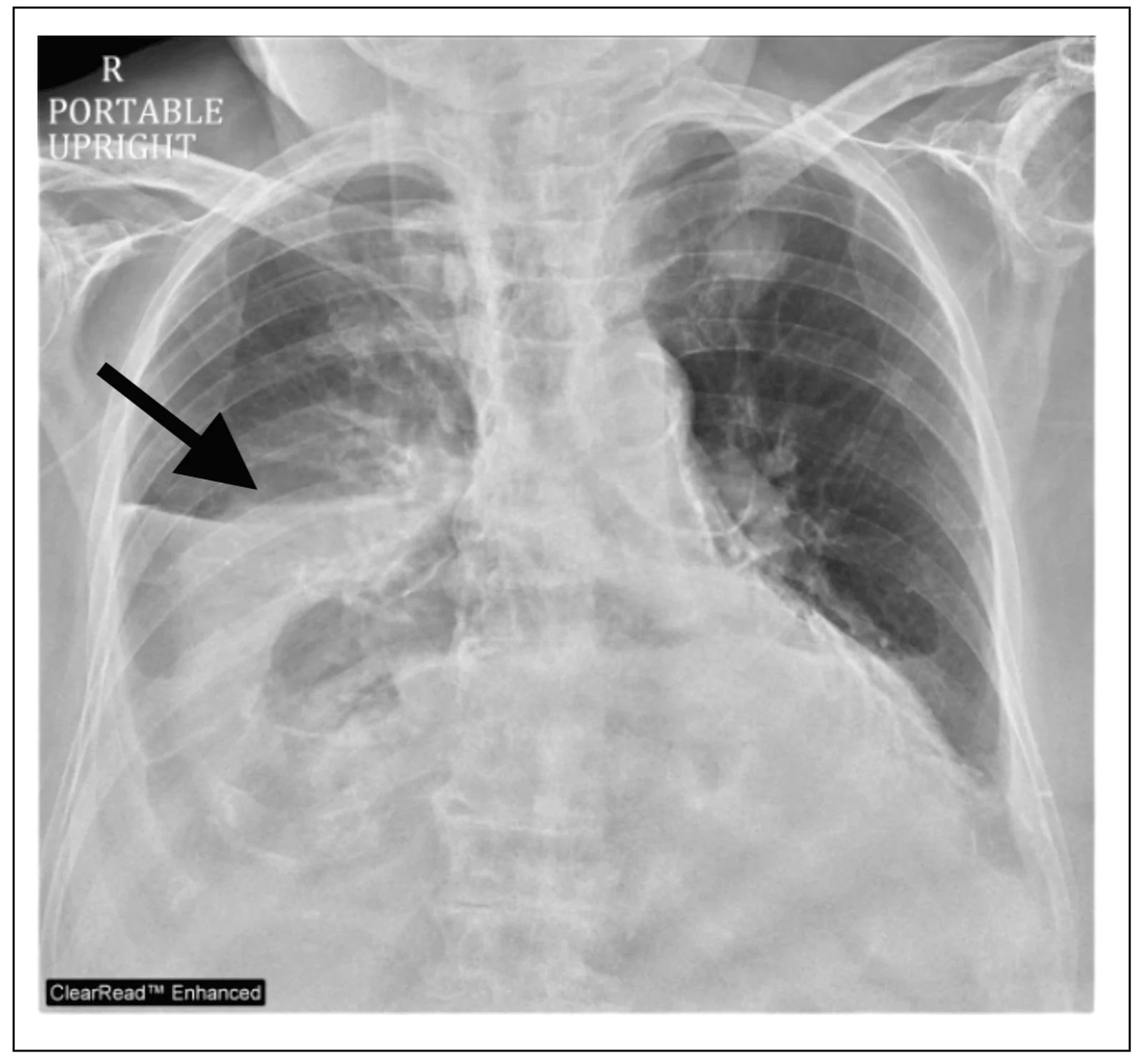
Chest X-ray showing effects of hepatomegaly on the thoracic cavity The chest radiograph was processed with ClearRead Xray Enhance (Riverain Technologies, Miamisburg, USA) to optimize contrast.

The patient was referred to an oncologist who treated her with atezolizumab and bevacizumab. The patient received a total of four cycles, with each cycle three weeks long. As a result, the tumor shrank by approximately 50% in size, which was deemed a successful clinical response. However, after the fourth cycle, the patient noticed pain and weakness in her neck. These symptoms progressed over the next few days, and she was seen in the emergency room. Imaging of her neck and cervical spine was unremarkable, and she was discharged home on non-steroidal anti-inflammatory medication and a referral to physical therapy. A week later, her symptoms escalated to the point where she could no longer lift her head, and she began to have trouble swallowing. She was again seen in the emergency room and evaluated by a neurologist who hospitalized her for further testing. This further testing was consistent with myasthenia gravis (with an elevated acetylcholine level of 0.89 nmol/L), systemic myositis (elevated CRP 1.0 mg/dL), and myocarditis (peak troponin 1011 pg/mL), which are the components of TMOS. She was treated with intravenous methylprednisolone and then oral prednisone, as well as intravenous immunoglobulins (IVIGs). She showed clinical improvement over the next several days and was eventually transferred to a rehab facility. The patient’s lab results during this admission are shown in Table 1. A week later, she had a sudden clinical decline indicated by dysphagia and cervical muscle weakness. Subsequently, the patient was re-hospitalized and again treated with IVIGs with good clinical response. She was subsequently transferred back to the rehab facility for further rehabilitation. The ICI-based regimen (atezolizumab along with bevacizumab) was discontinued. Imaging of the abdomen showed a 50% reduction in the size of the hepatic tumor.

**Table 1:** Laboratory results on admission and discharge WBC: white blood cell, HGB: hemoglobin, PLT: platelet, eGFR: estimated glomerular filtration rate, AST: aspartate aminotransferase, ALT: alanine aminotransferase, CK: creatine kinase

Biochemical Marker	Admission	Discharge	Normal Range
Sodium	143 mmol/L	139 mmol/L	136-146 mmol/L
Potassium	4.4 mmol/L	3.8 mmol/L	3.5-5.1 mmol/L
Creatinine	0.91 mg/dl	1.0 mg/dL	0.50-1.00 mg/dL
Anion gap	13 mmol/L	11 mmol/L	2-13 mmol/L
WBC	7.2 K/uL	5.7 K/uL	4.0-10.0 K/uL
HGB	14 g/dL	13.3 g/dL	12.0-16.0 g/dL
PLT	200 K/uL	160 K/uL	150-400 K/uL
eGFR	61 mL/min/1.73m^2^	54 mL/min/1.73m^2^	≥60 mL/min/1.73m^2^
AST	96 U/L	88 U/L	<34 U/L
ALT	92 U/L	81 U/L	10-49 U/L
CK	464 U/L	302 U/L	26-180 U/L
Albumin	3.4 g/dL	3.3 g/dL	3.5-4.9 g/dL
Alkaline phosphatase	119 U/L	120 U/L	40-116 U/L

## Discussion

Immunotherapy has helped make significant improvements in the treatment of advanced forms of cancer [1]. Immune checkpoint inhibitors have been at the forefront of treatment improvement, as they block proteins that cancer cells use to hide from T cells. This process allows the body’s T cells to more effectively target cancer cells. ICIs can be associated with mild side effects such as fatigue, rashes, nausea, or diarrhea [2]. However, more severe side effects can also include neurologic complications such as migraines and neuropathies [3]. More serious types of neurologic complications can also be noted, such as encephalitis, Guillain-Barré syndrome, aseptic meningitis, transverse myelitis, and myasthenia gravis [4]. Some cases of myasthenia gravis can be associated with concomitant inflammatory myositis and myocarditis, a condition known as triple M overlap syndrome [5]. This condition can be extremely serious with a high mortality rate [6]. Complications from ICIs usually develop within the first three months of treatment [7]. Prompt consultation with a neurologist should be obtained, as early treatment initiation is important to limit the severity of the complications [8,9].

Cardiovascular toxicity can also develop with ICIs alongside neurotoxicity [10]. These complications can include pericarditis, arrhythmias, venous thromboembolism, vasculitis, or myocarditis [11]. Most of the patients have a history of prior cardiovascular risk factors [12]. Myocarditis, in particular, has a high mortality rate [10]. Such complications can occur after the first treatment cycle and are believed to be more common when a combination immunotherapy regimen is used [13,14]. High-dose glucocorticoids are generally used in these situations due to the high risk of cardiac complications. Unfortunately, some of these cases progress despite aggressive treatment, and cardiac transplantation may need to be considered [14]. Diaphragmatic involvement can complicate these situations, further compromising cardiopulmonary status [15]. Further immunosuppression with agents such as mycophenolate, abatacept, or anti-thymocyte globulin may be needed in serious cases [16].

The third component of the triad is systemic inflammatory myopathy. Both polymyositis and dermatomyositis are potential complications of ICI therapy [17]. Symptoms tend to begin after the second or third cycle of therapy [18]. The muscle involvement can be extensive, involving proximal muscles of the extremities, as well as facial and oculo-bulbar muscles [18,19]. Two clinical points are that myalgias can be a presenting symptom that can occur before clinical weakness, and a rash is rarely seen in these situations [19,20]. Diagnosis is based on clinical history, physical examination findings, and blood tests such as a creatine phosphokinase (CPK) test [20]. Electromyography (EMG) and nerve conduction velocity (NCV) testing can help in the diagnosis along with autoantibodies such as acetylcholine receptor antibodies, striational antibodies, and anti-transcription intermediary factor 1-gamma. Muscle biopsy testing is usually not necessary to make the diagnosis. As with the neurologic and cardiac complications, inflammatory myopathies are treated with glucocorticoids [18]. IVIGs can be used in severe cases, along with mycophenolate, tacrolimus, and plasmapheresis [18,19].

## Conclusions

While immune checkpoint inhibitors have dramatically improved treatment options while minimizing side effects for many patients facing cancer, it is important to recognize, quickly diagnose, and treat those who present with severe complications. One such complication is the so-called “triple M overlap syndrome”, where patients present with myasthenia gravis, myocarditis, and inflammatory myopathy. In this report, we discussed a case of an 88-year-old female patient who developed this complication. Clinicians should note the importance of early diagnosis of this condition to improve the likelihood of positive clinical outcomes. Though TMOS is somewhat rare, this should be considered as part of the differential to support early diagnosis. The patient was treated with high-dose steroids and several rounds of IVIG and improved over time. She was discharged to the rehabilitation center with the plan that she would likely require several months of intensive physical therapy to have a chance to return to her prior baseline.
